# Supplementary material for: Amelioration of functional and histopathological consequences after spinal cord injury through phosphodiesterase 4D (PDE4D) inhibition
Source: Neurotherapeutics. 2024 May 16;21(4):e00372. doi: 10.1016/j.neurot.2024.e00372 (PMC11284540; doi:10.1016/j.neurot.2024.e00372)
Supplement: Multimedia component 1 [file mmc1.docx]

**Supplementary Figures**

**Title:**
Amelioration of functional and histopathological consequences after spinal cord injury through phosphodiesterase 4D (PDE4D) inhibition

**Authors:**

Melissa Schepers^a,b,c,1^, Sven Hendrix^d,1^, Femke Mussen^a,b,e,1^, Elise van Breedam^f^, Peter Ponsaerts^f^, Stefanie Lemmens^e^, Niels Hellings^c,e^, Roberta Ricciarelli^g,h^, Ernesto Fedele^g,i^, Olga Bruno^j^, Chiara Brullo^j^, Jos Prickaerts^k^, Jana Van Broeckhoven^c,e,2^ , Tim Vanmierlo^a,b,c,2*^

^a^Department of Neuroscience, Biomedical Research Institute, Faculty of Medicine and Life Sciences, Hasselt University, 3500 Hasselt, Belgium
^b^Department of Psychiatry and Neuropsychology, School for Mental Health and Neuroscience, Maastricht University, 6229ER Maastricht, Netherlands
^c^University MS Centre (UMSC) Hasselt – Pelt, Belgium
^d^Institute for Translational Medicine, Medical School Hamburg, 20457 Hamburg, Germany
^e^Department of Immunology and Infection, Biomedical Research Institute, Faculty of Medicine and Life Sciences, Hasselt University, 3500 Hasselt, Belgium
^f^Laboratory of Experimental Hematology, Vaccine and Infectious Disease Institute (Vaxinfectio), University of Antwerp, 2610 Wilrijk, Belgium
^g^IRCCS Ospedale Policlinico San Martino, 16100 Genoa, Italy
^h^Department of Experimental Medicine, Section of General Pathology, University of Genoa, 16100 Genoa, Italy
^i^Department of Pharmacy, Section of Pharmacology and Toxicology, University of Genoa, 16100 Genoa, Italy
^j^Department of Pharmacy, Section of Medicinal Chemistry, University of Genoa, 16100 Genoa, Italy
^k^Peitho Translational, 6229ER Maastricht, The Netherlands

* Corresponding author.

^1^ Equally contributing first authors
^2^ Equally contributing last authors

Corresponding author: Tim Vanmierlo, [t.vanmierlo@maastrichtuniversity.nl](mailto:t.vanmierlo@maastrichtuniversity.nl)

**Supplementary figures**

**
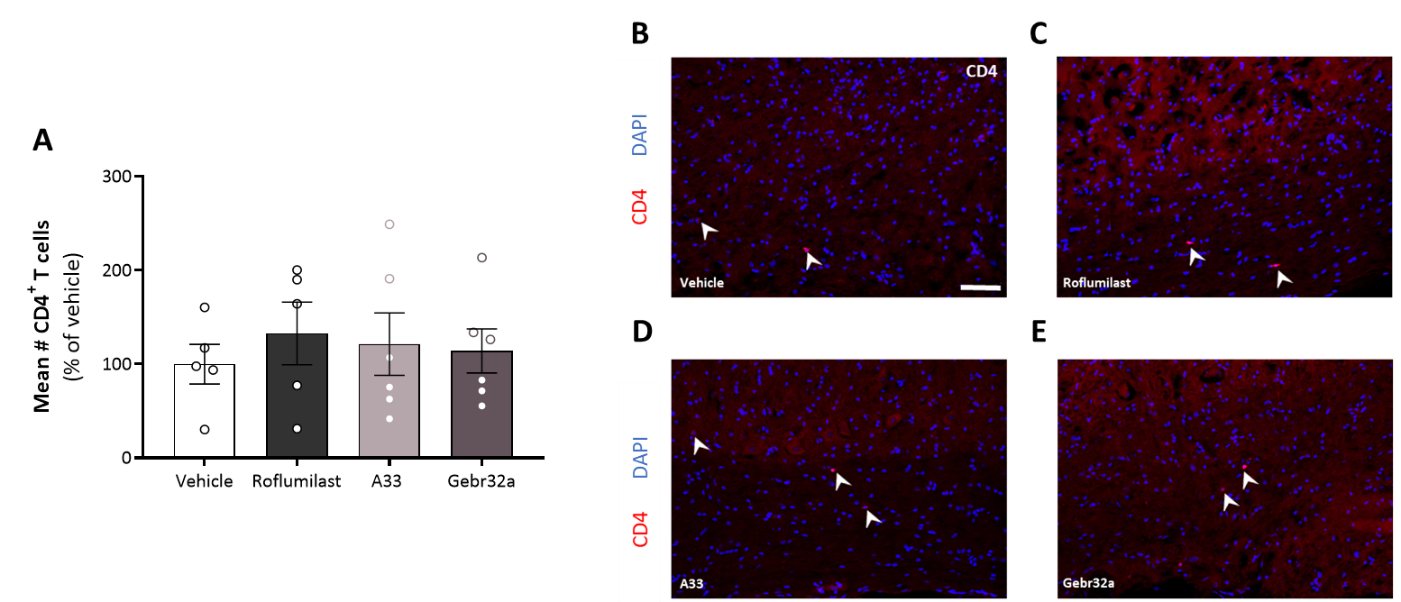
**

**Fig. S1 After spinal cord injury, the number of CD4^+^ T cells in the spinal cord does not change upon treatment with roflumilast, A33, or Gebr32a**. **(A-E)** Starting 1h after injury, mice were treated with vehicle, a general PDE4 inhibitor roflumilast (3 mg/kg), or gene-specific PDE4 inhibitors, A33 (3 mg/kg) and Gebr32a (0.3 mg/kg). **(A)** CD4 staining in spinal cord sections revealed no changes in the number of CD4^+^ T cells between the different groups. *n* = 5-6 mice/group. **(B-E)** Representative images of the CD4^+^ cells in the spinal cord sections of mice treated with the different PDE4 inhibitors. White arrows indicate the cells. Scale bar = 75 µm. Results were analyzed using a one-way ANOVA with Dunnett’s multiple comparison test (compared to vehicle). Data are displayed as mean +/-SEM

**
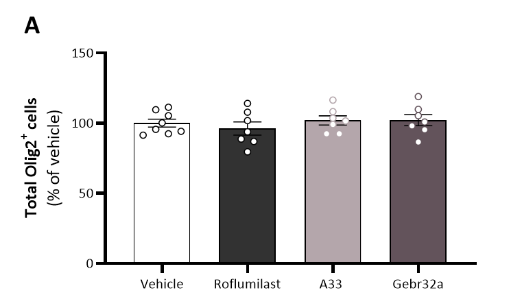
**

**Fig. S2 After spinal cord injury, the total number of Olig2^+^ oligodendrolineage cells does not change upon roflumilast, A33 or Gebr32a treatment. (A)** Starting 1h after injury, mice were treated with vehicle, a general PDE4 inhibitor roflumilast (3 mg/kg), or gene-specific PDE4 inhibitors, A33 (3 mg/kg) and Gebr32a (0.3 mg/kg). Olig2 staining in spinal cord sections revealed no differences in total number of oligodendrolineage cells between the different treatment groups. *n* = 7-8 mice/group. Results were analyzed using a one-way ANOVA with Dunnett’s multiple comparison test (compared to vehicle). Data are displayed as mean +/-SEM
